# Age Differences of Salivary Alpha-Amylase Levels of Basal and Acute Responses to Citric Acid Stimulation Between Chinese Children and Adults

**DOI:** 10.3389/fphys.2015.00340

**Published:** 2015-11-18

**Authors:** Ze-Min Yang, Long-Hui Chen, Min Zhang, Jing Lin, Jie Zhang, Wei-Wen Chen, Xiao-Rong Yang

**Affiliations:** ^1^Department of Biochemistry and Molecular Biology, School of Basic Courses, Guangdong Pharmaceutical UniversityGuangzhou, China; ^2^Pi-Wei Institute, Guangzhou University of Chinese MedicineGuangzhou, China; ^3^Pediatrics of Traditional Chinese Medicine, Haizhu Maternal and Child Health HospitalGuangzhou, China; ^4^Clinical Laboratory, The First Affiliated Hospital of Guangdong Pharmaceutical UniversityGuangzhou, China

**Keywords:** salivary alpha-amylase, *AMY1* copy number, protein expression, glycosylation, citric acid stimulation, children, adults

## Abstract

It remains unclear how salivary alpha-amylase (sAA) levels respond to mechanical stimuli in different age groups. In addition, the role played by the sAA gene (*AMY1*) copy number and protein expression (glycosylated and non-glycosylated) in sAA activity has also been rarely reported. In this study, we analyzed saliva samples collected before and after citric acid stimulation from 47 child and 47 adult Chinese subjects. We observed that adults had higher sAA activity and sAA glycosylated levels (glycosylated sAA amount/total sAA amount) in basal and stimulated saliva when compared with children, while no differences were found in total or glycosylated sAA amount between them. Interestingly, adults showed attenuated sAA activity levels increase over those of children after stimulation. Correlation analysis showed that total sAA amount, glycosylated sAA amount, and *AMY1* copy number × total sAA amount were all positively correlated with sAA activity before and after stimulation in both groups. Interestingly, correlation r between sAA levels (glycosylated sAA amount and total sAA amount) and sAA activity decreased after stimulation in children, while adults showed an increase in correlation r. In addition, the correlation r between *AMY1* copy number × total sAA amount and sAA activity was higher than that between *AMY1* copy number, total sAA amount, and sAA activity, respectively. Taken together, our results suggest that total sAA amount, glycosylated sAA amount, and the positive interaction between *AMY1* copy number and total sAA amount are crucial in influencing sAA activity before and after stimulation in children and adults.

## Introduction

Salivary alpha-amylase (sAA) is an endo-enzyme that catalyzes the hydrolysis of α-1, 4 glycosidic linkages of starch to produce maltose, maltotriose, and larger oligosaccharides. It is one of the most important enzymes in saliva. Recent studies have shown that sAA activity and amount could influence individual oral perception and intake of dietary starch, and can further affect overall nutritional status (Mandel et al., 2010; Chen et al., 2015). Secretions of sAA begin in the mouth by the salivary glands, which are innervated by efferent sympathetic nerves. Interestingly, sAA is not present in the oral compartment at birth, (O'Donnell and Miller, 1980) and sympathetic innervation of the salivary glands develops postnatally (Knox and Hoffman, 2008). Furthermore, studies have shown that the morphology of oral mucosa, functions of salivary glands, and saliva compositions all change with age (Ghezzi and Ship, 2003). Hence, sAA production shows developmental differences.

Basal sAA activity with regards to age difference appears to increase from undetectable concentrations in newborns to levels similar to adult concentrations at adolescence before growing up (Ben-Aryeh et al., 1984, 1990; Dezan et al., 2002; Davis and Granger, 2009; Stroud et al., 2009), but sAA does not change over the adult life span and remains constant even in older age (Pajukoski et al., 1997; Salvolini et al., 1999; Strahler et al., 2010). This might be related to the physiological development of salivary glands. Human salivary glands complete morphologically *in utero* and their growth continues in childhood by proliferation of well-differentiated cells (Redman, 1987). These glands complete developmental processes and mature in adulthood. Although a loss of acinar cells occurs with aging, salivary production remains age-stable in healthy adults due to a secretory reserve capacity of major salivary glands (Ghezzi and Ship, 2003). Divergent data was obtained from previous studies that directly compared basal sAA activity between children and adults. One study reported significant decrease in sAA activity in children (ages 9–12 years) when compared with adults (ages 18–23 years) (Yim et al., 2010), while other investigators found opposite results (Sivakumar et al., 2009; Strahler et al., 2010). Moreover, Ben-Aryeh et al. showed no significant difference between children (ages 6–8 years) and adults (ages 25–63 years) (Ben-Aryeh et al., 1990). One reason explaining the divergence might be related to age-based grouping differences of children and adults among these studies. The other reason might be related to different genetic or ethnic backgrounds of subjects in these studies. Studies have shown that Caucasian children with European background had higher salivary protein levels than those with African background (Sivakumar et al., 2009), and populations with high-starch diets had higher sAA protein levels than those traditionally fed on low-starch diets (Perry et al., 2007).

Salivary glands secret sAA mainly in response to beta-adrenergic stimuli (Chatterton et al., 1996). Numerous studies have shown that psychosocial and physical stressors can rapidly increase sAA activity. Hence, sAA has been proposed as a potential non-invasive biomarker for activity of the sympathetic nervous system (SNS) (Nater and Rohleder, 2009).

Age differences in acute sAA responses to stress have also been fully investigated from infants to adults in recent years. Increases in sAA to well-baby exam/inoculation in infants were evident at 6 and 12 months, but not at 2 or 24 months of age (Davis and Granger, 2009). Children (ages 7–12 years) showed an attenuated sAA response to a performance stress and peer rejection when compared with adolescents (ages 13–17 years) (Stroud et al., 2009). Interestingly, Strahler et al. found that sAA response to the Trier Social Stress Test (TSST) significantly increased across the following age groups: Children (ages 6–10 years), young adults (ages 20–31 years), and older adults (ages 59–61 years), while children and older adults showed lower sAA response relative to young adults (Strahler et al., 2010). In contrast, sAA increases to both an identical psychosocial laboratory stressor and a recall interview were not detected among children (ages 9–12 years) (Yim et al., 2010), and no age differences were found in sAA responses to the TSST between younger adults (ages 18–35 years) and older adults (54–71 years) (Almela et al., 2011). These divergent results might be related to the nature of the task or type of stressor and cognitive levels of subjects. Several studies had suggested that sAA levels were associated with participants' cognitive abilities (Willoughby et al., 2010; Keller et al., 2012), and cognitive effort was considered one important variable in the stress response study (Yim et al., 2010). A recent study showed that a non-stressful “placebo version” of the TSST required cognitive effort from participants (Het et al., 2009). Hence, more research is needed to determine how different types of stressors influence sAA response across different age groups (Yim et al., 2010).

Taken together, it showed high variability in both basal sAA activity and sAA responses to stress among different age groups. This is due to a number of physiological, psychosocial, and environmental factors, such as salivary gland development (Redman, 1987; Ghezzi and Ship, 2003; Knox and Hoffman, 2008), stress responses involving the autonomic nervous system (ANS) and the hypothalamus–pituitary–adrenal (HPA) axis (Davis and Granger, 2009), circadian rhythms (Nater et al., 2007), stress levels (Stroud et al., 2009; Strahler et al., 2010; Yim et al., 2010; Almela et al., 2011), and even eating habit (Squires, 1953; Perry et al., 2007). In addition, there was evidence showing that a stress-related Catechol-O-methyltransferase (COMT) functional polymorphism involving a substitution of valine (Val) by methionine (Met) at codon 158 (Val-^158^-Met), a serotonin transporter-linked promoter region (5-HTTLPR) polymorphism involving an insertion/deletion in the promoter region of the serotonin transporter gene, a Gamma-Aminobutyric Acid (GABA) A Receptor, and an Alpha 6 (GABRA6) polymorphism involving a single-nucleotide substitution from T to C at nucleotide 1521 in the 3′ untranslated region of the GABRA6 receptor gene, all played important role in determining individual differences in sAA responses to stress (Zubieta et al., 2003; Uhart et al., 2004; Frigerio et al., 2009).

Genetically, sAA is coded by the *AMY1* gene, which is one of the most variable copy number variation (CNV) loci in the human genome. The *AMY1* gene copy number was reported to be within a range of 1 to 15 diploid copies (Bank et al., 1992). Perry et al. (2007) and Mandel et al. (2010) found that the *AMY1* copy number was positively correlated with sAA protein expression in humans, and that sAA the amount was also positively correlated with its activity. About 25–30% of human sAA was glycosylated after being secreted into the oral cavity (Keller et al., 1971). Previous studies have found that N-glycosylation levels affected sAA secretion, its enzyme activity and stability (de Barros et al., 2009), and that β-adrenergic stimulation increased N-linked protein glycosylation (Kousvelari et al., 1984). Hence, we hypothesize that *AMY1* copy number variations, sAA protein amount, and its glycosylated levels may be important biological factors leading to sAA activity variation among different age groups.

Chinese people have been mainly consuming high-starch (e.g., rice) diet for thousands of years so that they are undoubtedly categorized as high-starch population. Starch-based foods are expected to provide most of the energy sources for their daily life. sAA is mainly involved in the initiation of the digestion of starch in the oral cavity. Mandel et al. have recently found that sAA levels could influence the oral perception of textural attributes of starchy foods and thus might determine individual's liking and preference for a starchy food (Mandel et al., 2010). Childhood is a sensitive period of growth and development so that children may be more susceptible to environmental changes. For example, changes in oral perception may significantly influence child's liking and preference for foods. Our previous work further indicated that sAA levels might partially determine individual's nutritional status in Chinese children (Chen et al., 2015). However, the nutritional status of adults has been shaped by combined actions of innate and environmental factors. So we expect it would be of importance to compare sAA levels and responses between children and adults. To date, however, rare studies have investigated sAA levels and responses with Chinese subjects across different age groups.

In summary, previous studies of sAA activity and responses related to age differences had some of the following characteristics: (1) divergent data in investigating sAA activity of basal and acute responses to stress between children and adults, (2) a multitude of investigations focused on the possibility of sAA as a potential biomarker of SNS activity, while less attention was drawn to the contributions of *AMY1* gene CNVs and sAA protein expression and modifications to the variability of sAA responses to stress among different age groups, (3) many psychosocial stressors were introduced (e.g., TSST and performance stressor), while mechanical stimuli (e.g., citric acid) were rarely studied, and (4) rare Asian populations were studied.

We set out in the present study to investigate sAA activity in basal and acute response to citric acid stimulation between Chinese children and adults. We also measured the *AMY1* gene copy number and total (glycosylated and non-glycosylated) and glycosylated sAA amount, which may act as important biological factors influencing sAA activity of basal and acute responses. The goals of this study were to look at the basal and acute responses of sAA activity to citric acid stimulation, the *AMY1* copy number, the total/glycosylated sAA amount, and to further assess developmental changes and secretion patterns of salivary glands between children and adults.

## Materials and methods

### Ethics statement

The present study was conducted according to the Declaration of Helsinki and approved by the Academic Ethics Committee of Guangdong Pharmaceutical University. Written informed consent was obtained from all adult participants. All children were accompanied by at least one of their parents or guardians, who also signed the informed consent and stayed throughout the saliva collection.

### Participants

Children aged 6–10 and adults aged 20–25 were recruited from Haizhu Maternal and Child Health Hospital of Guangzhou and Guangdong Pharmaceutical University, respectively. Participants were excluded if they presented dry mouth, salivary gland diseases, oral diseases, neurogenic diseases, or facial asymmetry. Participants were also excluded if they were currently taking asthma medications, anti-rheumatic medications, any psychotropic substances, sleeping pills, or painkillers (Rohleder and Nater, 2009; Strahler et al., 2010). Adult females were excluded if they regularly took oral contraceptives, and young females were also excluded if they already had menarche. Participants were free of psychiatric and severe somatic diseases as determined by an interview by two of the authors; LHC (responsible for the interview of adults) and MZ (responsible for the children). All participants denied smoking, alcohol abuse, and acute caffeine consumption. In the end, 47 children (24 Females, 23 Males) and 47 adults (24 Females, 23 Males) fulfilled the inclusion criteria.

### Stimuli

We used a standard stimulus, which was first used by Pi-Wei Institute of Guangzhou University of Chinese Medicine in 1978. Pieces of filter paper of fixed size (1 × 1 cm) were soaked in 0.4 mol/L citric acid solution for 10 min, dried in a drying oven, then collected and stored in a clean container until their use.

### Saliva collection, handling, and storage

Saliva collection was limited to between the hours of 09.00 and 11.00 in the morning to minimize the effect of diurnal variations (Nater et al., 2007). Subjects were not allowed to eat or drink anything but water for 1 h before saliva collection. Saliva collection was carried out according to our previous description (Chen et al., 2013, 2015). After a 30-min resting period to minimize the impacts of physical activity and emotion, subjects were instructed to be seated with eyes open, head slightly forward, and to empty their mouths by swallowing all saliva. The unstimulated saliva was obtained by passive drooling into 5 mL test tubes for 3 min (without tongue movements). Salivary secretion was then stimulated by placing a 1 × 1 cm filter paper containing citric acid on the tip of each participant's tongue for 1 min, during which the stimulated saliva was collected from under the tongue into a new 5 mL test tube. Participants were asked to keep their tongue tips slightly upward when collecting stimulated saliva, so that the citric acid in the filter paper would not mix with the collected saliva. The pH values of all saliva samples were determined before storage by precision pH test paper with a range from 6.0 to 8.0. All saliva samples went through one freeze-thaw cycle to break down mucopolysaccharides that could interfere with pipetting (Shirtcliff et al., 2001). Upon thawing at 4°C, the saliva samples were centrifuged at 1, 1000 × g at 4°C for 10 min. The supernatant was aliquoted and stored at −80°C for subsequent measurements of sAA activity and amount. The sediment (containing cheek cells) was frozen at −80°C for DNA analysis.

### Enzymatic activity assay for sAA

According to the fact that salivary and pancreatic alpha-amylase share 97% homology, sAA activity was determined using a kinetic reaction assay kit (KOFA Biotech Company, Guangzhou, China) on a Hitachi 7180 automatic biochemical analyzer in the clinical laboratory of the First Affiliated Hospital of Guangdong Pharmaceutical University. Saliva samples were diluted to 1/200th their original concentration, and then incubated with a specific chromogenic substrate, 4,6-ethyliden-G7-PNP, and the auxiliary enzyme α-glucosidase. The substrate was first cleaved by alpha-amylase and then by auxiliary enzyme into p-nitrophenol (PNP), which had light absorption peak at 405 nm (yellow). The absorbance value of PNP at 405 nm was directly proportional to the alpha-amylase activity. Knowing this, sAA activity was determined by measuring the light absorbance of PNP, and was expressed as U/mL. Coefficient variations (CV) of intra-assay from nine replicates and inter-assay from 10 separate samples were less than 5%.

### SDS-PAGE and immunoblotting for sAA

Before SDS-PAGE, total protein content of all saliva samples was determined using a bicinchoninic acid Protein Assay Kit (Beyotime, Shanghai, China). The immunoblotting experiments were performed in a fashion similar to that described by Perry et al. (2007). Saliva samples of equal quantity protein (5 μg) were prepared by solubilizing samples in SDS-PAGE sample loading buffer and heating to 100°C for 5 min. For quantification purpose, a human sAA protein sample (Sigma-Aldrich, St. Louis, MD, USA) of known quantity was run on each gel. Proteins were separated by SDS-PAGE (5% stacking gel and 10% separation gel) and transferred onto a polyvinylidene fluoride (PVDF) membrane (Roche, Shanghai, China) in transfer buffer for 70 min at 350 mA. Membranes were blocked for 2 h at room temperature in blocking buffer (PBS, 0.1% tween-20) with 5% milk. Membranes were then incubated overnight at 4°C with sheep anti-alpha-amylase (Abcam, HK, China), diluted 1:5000 in blocking buffer. After washing in phosphate-buffered saline tween-20 (PBST), membranes were incubated for 2 h in donkey anti-sheep IgG-horseradish peroxidase conjugate (R&D Systems, Minneapolis, MN, USA), and diluted 1:1000 in blocking buffer. Membranes were washed again and exposed to 3,3′-diaminobenzidine (DAB) substrate (Tiangen Biotech, Beijing, China) for 2 min. The G: BOX HR gel documentation system (Syngene, Cambridge, UK) was used for detection of amylase. Quantification of protein bands was performed using Gel-Pro Analyzer 4.0 software (Media Cybernetics, Rockville, MD, USA). The total and glycosylated sAA amounts of test samples were estimated by comparing human sAA of known quantity. The glycosylated sAA levels were determined using relative proportion of glycosylated sAA to total sAA.

### DNA extraction and quantitative PCR for the *AMY1* gene

Saliva (containing cheek cells) is considered as a good source to perform DNA extraction due to the ease of sampling (Nemoda et al., 2011). In the present study, genomic DNA was extracted from whole saliva according to our previous methods (Chen et al., 2015). Briefly, the frozen salivary sediment (containing cheek cells) was thawed at 4°C, then washed and scattered using 0.01 mol/L PBS. Cheek cells were collected by centrifugation at 11,000 × g for 10 min. Cell lysis was achieved by adding 50 μL 5 mol/L KI solution and vortexing for 30 s. 100 μL 0.9% NaCl solution and 150 uL chloroform were added to the lysate to precipitate proteins by vortexing for 30 s and then centrifugation at 11,000 g for 3 min. The supernatant was transferred into a new 1.5 mL eppendorf tube followed by adding an equal volume of isopropanol. After centrifugation at 11,000 g for 3 min, the DNA pellet was washed once in 500 μL ethanol and dried at room temperature. Finally, DNA was dissolved by adding 30 μL TE (10 mM Tris, 1 mM EDTA) buffer and then stored at −20°C. DNA quality and quantity were estimated by measuring the absorbance of the sample at wavelengths 260 nm and 280 nm using a NanoDrop spectrophotometer (Thermo Scientific, Wilmington, DE, USA). An optical density of 1 at 260 nm corresponds to about 50 ng/μL of double-stranded DNA, whereas 1.8 or higher 260/280 nm ratio indicates good DNA quality. The average concentration (ng/μL) of extracted DNA from 94 DNA samples was 266.88 ± 283.73, and average 260/280 nm ratio was 1.99 ± 0.15, which both agreed with the reports by Nemoda et al. (2011). For *AMY1* CNV analyses, DNA samples were standardized to a final concentration of 10 ng/μL.

Quantitative PCR (qPCR) was performed to determine diploid *AMY1* gene copy number according to the methods of Perry et al. (2007). Primers for *AMY1* and tumor protein p53 (*TP53*) gene amplification were also from the literature, and *TP53* was amplified to adjust for DNA dilution quantity variation. DNA samples were run in triplicate 20 μL volume reactions using iTaq Universal SYBR Green Supermix (Bio-Rad, Hercules, CA, USA) on a Bio-Rad CFX96 Touch Real-Time PCR Machine. For each test sample replicate, 10 ng of genomic DNA was used. Thermal cycling was organized in three repeated steps: the first denaturation step of 3 min at 95°C, followed by 39 repeated cycles of 95°C for 15 s and 60°C for 30 s. Data was analyzed using CFX Manager Software version 2.1 (Bio-Rad). *AMY1* diploid copy number was estimated using a standard curve constructed from the reference DNA sample (NA18972; Coriell Cell Repositories, Camden, NJ, USA), which was previously determined to have 14 *AMY1* diploid copies by qPCR and Fiber FISH (fluorescence *in situ* hybridization) (Perry et al., 2007; Mandel et al., 2010).

### Statistical analysis

Data was tested for normal distribution and homogeneity of variance using Kolmogorov-Smirnov and Levene's tests before statistical procedures were applied. These analyses revealed significant deviations of some sAA activity and amount values. Therefore, they were log transformed prior to analyses, which restored normality of distribution. Preliminary analyses were performed using *chi-square* and *t*-tests to investigate sex and age differences in the demographic variables, respectively, and *t*-tests were used to identify sex factors that might influence sAA levels. Student's un-paired *t*-tests was used to investigate age differences in sAA levels, *AMY1* copy number, and sAA activity ratio between children and adults, and paired *t*-tests were introduced to investigate the differences in sAA levels of basal and acute responses to citric acid stimulation within children and adults. Pearson correlation coefficient tests were used to examine the correlations between sAA activity, sAA amount, and *AMY1* copy number. All *p*-values reported were two-tailed, and the level of significance was marked at < 0.05. The results shown in the text were raw values to facilitate interpretation. Statistical analyses were performed using SPSS statistics version 20.0 (IBM Software, USA), and graphing was performed using GraphPad Prism 5.0 (GraphPad Software, La Jolla, CA, USA).

## Results

### Sample characteristics

Age differed significantly between child (7.6 ± 1.3, ranging from 6 to 10 years) and adult (22.4 ± 1.8, ranging from 20 to 25 years) groups (*t*_92_ = 45.03, *p* < 0.001), but did not differ significantly between males (children: 7.8 ± 1.5, ranging from 6 to 10 years; adults: 22.4 ± 1.9, ranging from 20 to 25 years) and females (children: 7.3 ± 1.0, ranging from 6 to 10 years; adults: 22.3 ± 1.7, ranging from 20 to 25 years) from within the two groups (children: *t*_45_ = 1.445, *p* = 0.155; adults: *t*_45_ = 0.263, *p* = 0.794). There were also no sex differences between them (children: 24 Females, 23 Males; adults: 24 Females, 23 Males) (χ^2^ = 0, *p* = 1.000). Furthermore, no sex-related differences of sAA levels were found within both age groups (all *p* > 0.10).

### Salivary amylase levels and *AMY1* copy number

**Figure 1:**
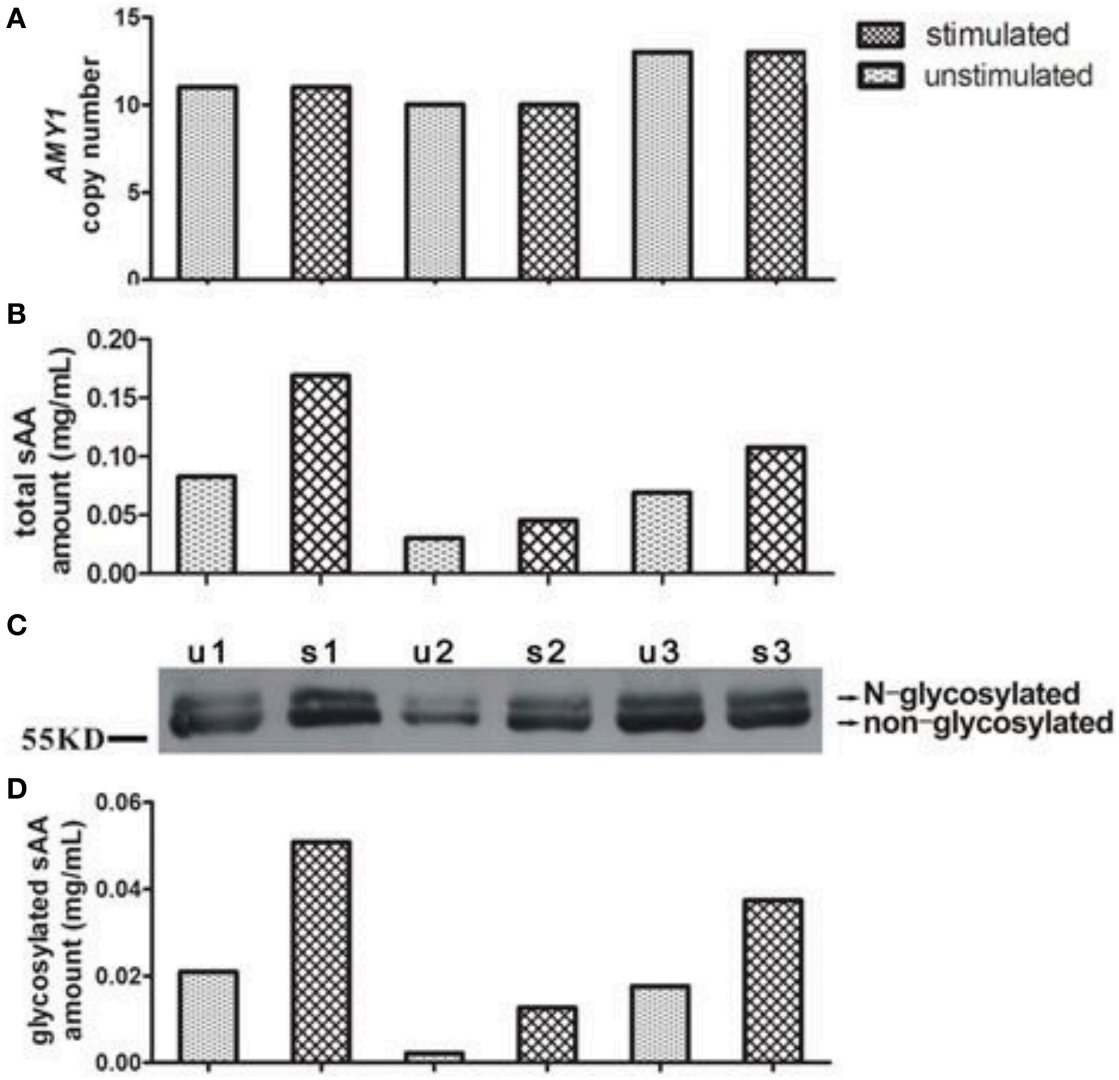
**Representative of variations of ***AMY1*** copy number, total and glycosylated sAA amount from three subjects**. The *AMY1* copy number **(A)** was estimated by qPCR. Total **(B)** and glycosylated **(D)** sAA amount of unstimulated (u) and stimulated (s) saliva were estimated by the method mentioned in Materials and Methods. A Western blot image **(C)** is served as representative.

In Figure 1, we showed representative of variations of *AMY1* copy number, total, and glycosylated sAA amount from three subjects. Measurements of sAA activity (U/mL), total, and glycosylated sAA amounts (mg/mL), sAA glycosylated levels, sAA activity ratio, and *AMY1* copy number were fully displayed in Table 1. Adults showed higher sAA activity (basal: *t*_92_ = −7.563, *p* < 0.001; stimulated: *t*_92_ = −5.028, *p* < 0.001) and sAA glycosylated levels (basal: *t*_92_ = −2.316, *p* = 0.023; stimulated: *t*_92_ = −2.090, *p* = 0.039) than that of children, while the two groups had no differences in total sAA amount (basal: *t*_92_ = −1.80, *p* = 0.857; stimulated: *t*_92_ = 1.277, *p* = 0.205) and glycosylated sAA amount (basal: *t*_92_ = 1.440, *p* = 0.154; stimulated: *t*_92_ = 0.431, *p* = 0.668). Upon citric acid stimulation, sAA activity (*t*_46_ = −6.043, *p* < 0.001), total sAA amount (*t*_46_ = −3.237, *p* = 0.002), glycosylated sAA amount (*t*_46_ = 4.420, *p* < 0.001), and sAA glycosylated levels (*t*_46_ = −3.727, *p* = 0.001) significantly increased in children, while adults only showed increase in glycosylated sAA amount (*t*_46_ = 2.107, *p* = 0.041) and sAA glycosylated levels (*t*_46_ = −4.219, *p* < 0.001). Moreover, adults showed markedly attenuated sAA activity responses to citric acid stimulation compared with children (*t*_92_ = 3.524, *p* = 0.001).

**Table 1:** **sAA levels (means ± SD) and ***AMY1*** copy number between children and adults**.

**Variable**	**Children group (*n* = 47)**			**Adults group (*n* = 47)**			***p*^b^**	
	**Unstimulated**	**Stimulated**	***p*^a^**	**Unstimulated**	**Stimulated**	***p*^a^**	**Unstimulated**	**Stimulated**
sAA activity (U/mL)	25.28(26.99)	42.20(35.36)	0.000	85.72(62.72)	89.71(53.94)	0.310	0.000	0.000
Total sAA amount (mg/mL)	0.14(0.22)	0.20(0.25)	0.002	0.10(0.07)	0.11(0.09)	0.765	0.857	0.205
Glycosylated sAA amount (mg/mL)	0.03(0.07)	0.05(0.07)	0.000	0.02(0.02)	0.03(0.03)	0.041	0.154	0.668
sAA glycosylated levels^c^	0.18(0.12)	0.24(0.09)	0.001	0.23(0.11)	0.28(0.10)	0.000	0.023	0.039
sAA activity ratio^d^	2.24(1.53)			1.42(1.26)			0.001	
*AMY1* copy number	7.9(2.5) (range from 2 to 15)			8.6(3.5) (range from 1 to 15)			0.253	

a *Student's paired t-test for before and after citric acid stimulation intra-groups of children and adults*.

b *Student's un-paired t-test for before and after citric acid stimulation inter-groups of children and adults*.

c *Proportion of glycosylated sAA amount in total sAA amount*.

d *Stimulated sAA activity/unstimulated sAA activity*.

The average numbers of *AMY1* gene copies were 7.9 (±2.5) and 8.6 (±3.5) in children and adults, respectively, and no difference was observed between them (*t*_92_ = −1.150, *p* = 0.253). Besides, *AMY1* copy number ranged from 2 to 15 in children, and 1 to 15 in adults, respectively. We combined both groups and found on average eight copies of *AMY1* (data not shown), which was higher than that of previous studies with low-starch populations (Perry et al., 2007; Mandel et al., 2010).

### Correlations between *AMY1* copy number, sAA activity, and sAA amount

In order to assess the roles played by *AMY1* copy number and sAA protein expression in sAA activity and acute sAA responses to citric acid stimulation between children and adults, we analyzed the correlation coefficients (r) among *AMY1* copy number, sAA activity, and sAA amount before and after citric acid stimulation.

**Figure 2:**
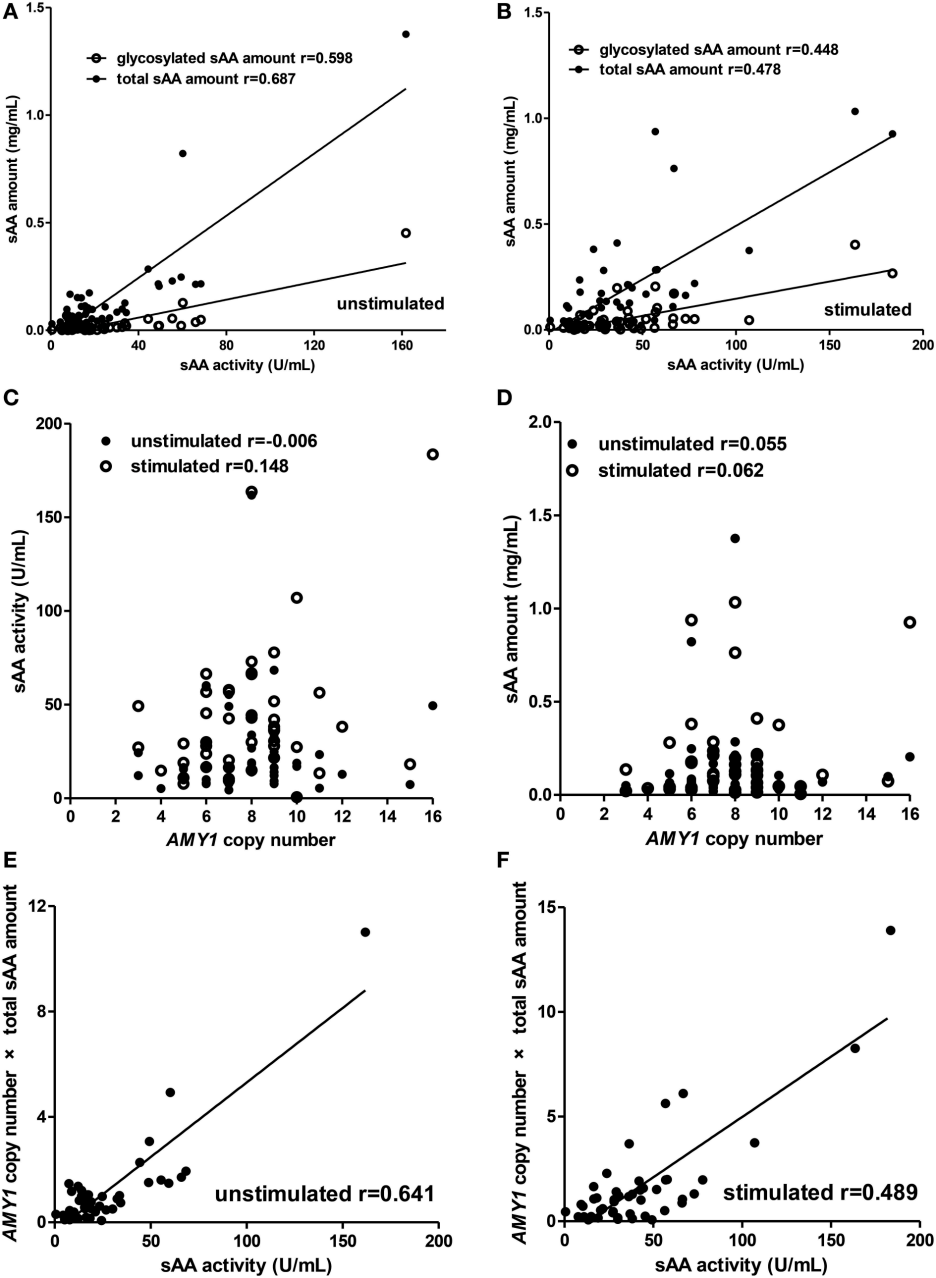
**Correlations among *AMY1* copy number, sAA activity, and sAA amount before and after citric acid stimulation in children**. Both total sAA amount (mg/mL) and glycosylated sAA amount (mg/mL) were significantly correlated with sAA activity (U/mL) in unstimulated **(A)** and stimulated saliva **(B)**. After stimulation, correlation r between glycosylated sAA amount, total sAA amount, and sAA activity decreased from 0.598 to 0.448 and from 0.687 to 0.478, respectively. Neither sAA activity **(C)** nor total sAA amount **(D)** was correlated with *AMY1* copy number in unstimulated or stimulated saliva. *AMY1* copy number × total sAA amount significantly correlated with sAA activity in unstimulated **(E)** and stimulated saliva **(F)**. Interestingly, the correlation r between *AMY1* copy number × total sAA amount and sAA activity was higher than that between *AMY1* copy number and sAA activity in unstimulated saliva, and also higher than that between *AMY1* copy number, total sAA amount, and sAA activity in stimulated saliva.

For the children group (Figure 2), total sAA amount (unstimulated: *r* = 0.687, *p* < 0.001; stimulated: *r* = 0.478, *p* = 0.001), glycosylated sAA amount (unstimulated: *r* = 0.598, *p* < 0.001; stimulated: *r* = 0.448, *p* = 0.002), and *AMY1* copy number × total sAA amount (unstimulated: *r* = 0.641, *p* < 0.001; stimulated: *r* = 0.489, *p* < 0.001) there showed positive correlations with sAA activity both in unstimulated and stimulated whole saliva (Figures 2A,B,E,F). Interestingly, we observed that correlation r between glycosylated sAA amount and sAA activity decreased from 0.598 to 0.448 after citric acid stimulation, and that correlation r between total sAA amount and sAA activity also decreased from 0.687 to 0.478. Moreover, the correlation r between *AMY1* copy number × total sAA amount and sAA activity (*r* = 0.641) was much higher that between *AMY1* copy number and sAA activity (*r* = −0.006) in unstimulated saliva, and also higher (*r* = 0.489) than that between total sAA amount (*r* = 0.478), *AMY1* copy number (*r* = 0.148) and sAA activity in stimulated saliva. However, neither sAA activity (Figure 2C) nor total sAA amount (Figure 2D) was significantly correlated with *AMY1* copy number in unstimulated or stimulated whole saliva (all *p* > 0.05).

**Figure 3:**
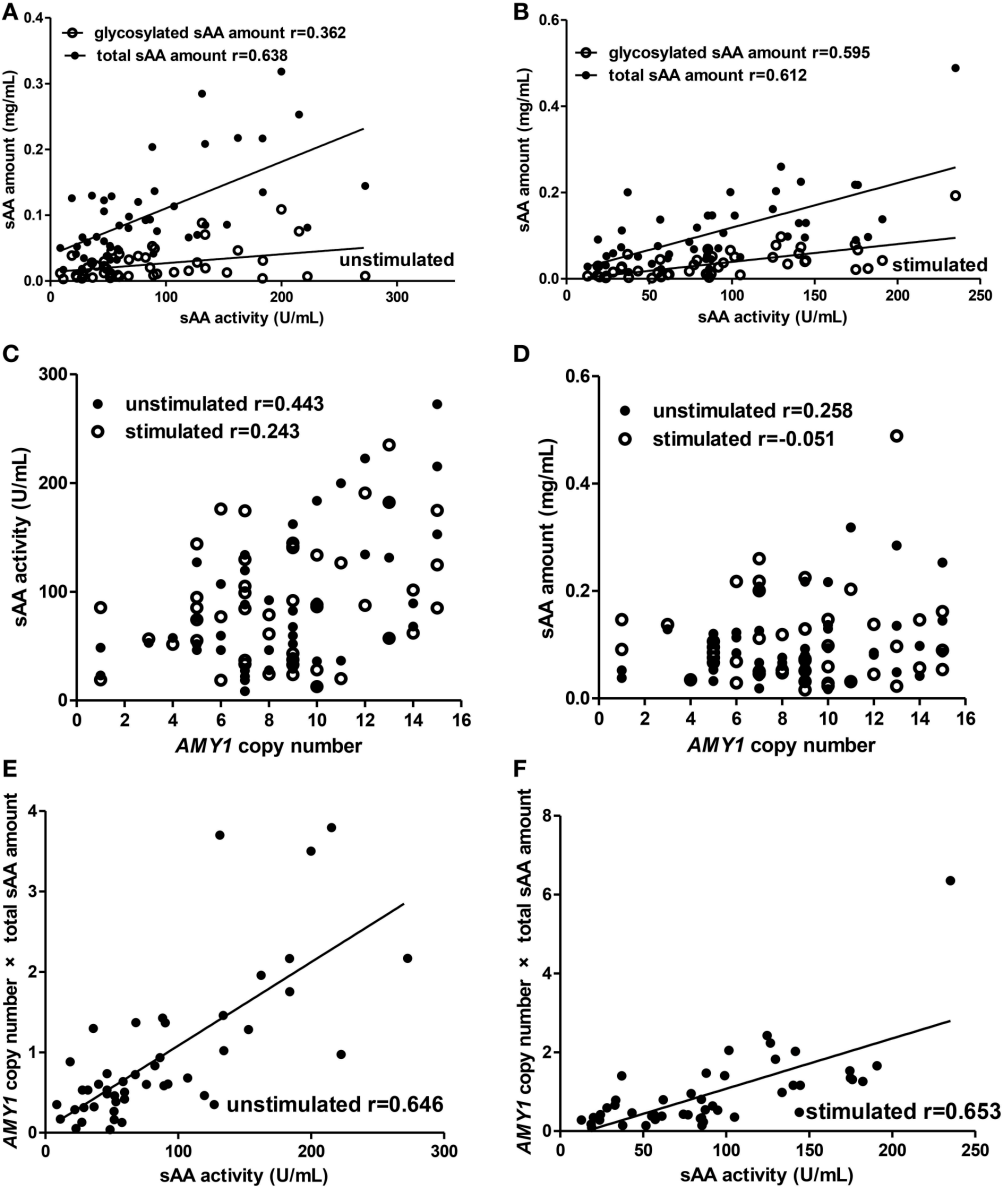
**Correlations among *AMY1* copy number, sAA activity, and sAA amount before and after citric acid stimulation in adults**. Both total sAA amount (mg/mL) and glycosylated sAA amount (mg/mL) were significantly correlated with sAA activity (U/mL) in unstimulated **(A)** and stimulated saliva **(B)**. Correlation r between glycosylated sAA amount and sAA activity increased from 0.362 to 0.595 after stimulation. Neither sAA activity **(C)** nor total sAA amount **(D)** was correlated with *AMY1* copy number in unstimulated or stimulated saliva, except that *AMY1* copy number was significantly correlated with sAA activity in unstimulated saliva (*r* = 0.443). *AMY1* copy number × total sAA amount significantly correlated with sAA activity in unstimulated **(E)** and stimulated saliva **(F)**. Interestingly, the correlation r between *AMY1* copy number × total sAA amount and sAA activity was higher than that between *AMY1* copy number, total sAA amount and sAA activity in unstimulated and stimulated saliva.

For the adults group (Figure 3), we observed similar results to children in the correlations between total sAA amount (unstimulated: *r* = 0.638, *p* < 0.001; stimulated: *r* = 0.612, *p* < 0.001), glycosylated sAA amount (unstimulated: *r* = 0.362, *p* = 0.014; stimulated: *r* = 0.595, *p* < 0.001), *AMY1* copy number × total sAA amount (unstimulated: *r* = 0.646, *p* < 0.001; stimulated: *r* = 0.653, *p* < 0.001) and sAA activity (Figures 3A,B,E,F). Interestingly, we observed correlation r between glycosylated sAA amount and sAA activity increased, rather than decrease, from 0.362 to 0.595 after stimulation. Moreover, the correlation r between *AMY1* copy number × total sAA amount and sAA activity (*r* = 0.646) was higher than that between total sAA amount (*r* = 0.638), *AMY1* copy number (*r* = 0.443) and sAA activity in unstimulated saliva, and also higher (*r* = 0.653) than that between total sAA amount (*r* = 0.612), *AMY1* copy number (*r* = 0.243) and sAA activity in unstimulated saliva. Besides, we found positive correlation between sAA activity and *AMY1* copy number in unstimulated (*r* = 0.443, *p* = 0.002) but not in stimulated (*r* = 0.243, *p* = 0.100) saliva (Figure 3C). Finally, no significant correlation was found between total sAA amount and *AMY1* copy number (Figure 3D).

## Discussion

In the present study, we directly compared basal and stimulated sAA activities between children and adults (which were categorized as high-starch population), and assessed for the first time the role played by *AMY1* copy number, total sAA amount, and glycosylated sAA amount in sAA activity with regards to age. We observed significant difference of sAA activity and sAA glycosylated level (glycosylated sAA amount/total sAA amount) in both basal and stimulated saliva between children and adults. Correlation analysis revealed that the total sAA amount, glycosylated sAA amount, and *AMY1* copy number × total sAA amount played important roles in influencing the age related differences in sAA activity, before and after stimulation. We also found attenuated acute sAA activity response to citric acid stimulation in adults compared with children. Finally, we reported on average eight copies of the *AMY1* gene, which was higher than that of previous studies of low-starch populations (Perry et al., 2007; Mandel et al., 2010).

Age related basal sAA activities and acute sAA responses to many kinds of stressors have been investigated in developmental- and psychological-related science (Ben-Aryeh et al., 1990; Pajukoski et al., 1997; Salvolini et al., 1999; Davis and Granger, 2009; Sivakumar et al., 2009; Stroud et al., 2009; Strahler et al., 2010; Yim et al., 2010; Almela et al., 2011). However, the results between children and adults remained divergent. In the present study, the passive drooling technique was introduced to collect saliva samples. This enabled researchers to collect basal whole saliva from all glands while minimizing possible mechanical stimulations (Navazesh, 1993). Hence, it was considered the best method for collection of unstimulated whole saliva (Rohleder and Nater, 2009). In addition, the stimulated whole saliva was collected after gustatory stimulation by citric acid (Froehlich et al., 1987; Chen et al., 2013, 2015). The final results showed that both basal and stimulated sAA activities were significantly higher in adults than that of children (*p* < 0.01). Although different types of stimulation and method of saliva collection were introduced, the findings were consistent with the reports by Yim et al. (2010). However, our findings disagreed with a study by Strahler et al. (2010), in which sAA activity significantly increased in children when compared to adults. Possible reasons for the divergent results might be due to genetic differences, ethnic differences, or age-based grouping differences of children and adults. Interestingly, we observed that children showed increased levels of sAA activity after citric acid stimulation, while adults showed attenuated sAA activity responses. These findings might disagree with the report by Stroud et al. (2009), in which children showed significantly attenuated sAA responses to peer rejection than adolescents. Moreover, our results might also disagree with the study by Strahler et al. (2010), in which significant sAA increases were found in three age groups (children, young adults, and older adults) but attenuated sAA responses to an acute laboratory stressor were observed in children when compared with young adults. One possible reason for all the divergent results might be due to the differences of stressor/stimulation type used to stimulate saliva secretion.

Few studies however, have investigated the contribution of sAA secretion patterns to sAA levels with regards to age. In the present study, we observed no difference of total sAA amount in basal or stimulated salvia between children and adults. However, adults showed higher sAA glycosylated levels than children in both basal and stimulated saliva. Combined with the finding that adults showed higher basal and stimulated sAA activity when compared to children, we argued that sAA glycosylated levels might play a more important role in determining sAA activity than total sAA amount. sAA was secreted from the salivary glands mainly in response to beta-adrenergic stimuli (Chatterton et al., 1996), which not only promoted sAA secretion, but also induced increased levels of N-linked protein glycosylation of sAA (Kousvelari et al., 1984). N-glycosylation levels affected sAA secretion, enzyme activity, and stability (de Barros et al., 2009). Previous studies have shown that glycosylated sAA had higher enzymatic activity than the non-glycosylated one (Koyama et al., 2000). Moreover, parotid, submandibular, and sublingual glands all responded differently to beta-adrenergic stimuli. Specifically, contributions of the parotid gland to whole saliva increased from 20% up to more than 50% after stimulation (Humphrey and Williamson, 2001). We found that sAA glycosylated levels in rat parotid glands were higher than that of submandibular or sublingual glands (to be published). Taken together, our results suggested sAA secretion patterns may be different between children and adults. In basal condition, children showed lower sAA activity than adults, which might be due to the possibility that children secreted relatively more non-glycosylated sAA from the submandibular and sublingual glands but less glycosylated sAA from the parotid glands compared with adults. Upon citric acid stimulation, contribution of the parotid glands toward sAA glycosylated levels significantly increased in both children and adults. However, adults showed attenuated sAA activity response, which might be due to a relatively decreased contribution of the parotid glands compared with children. Physiologically, childhood is a sensitive period of growth and development, which makes children more susceptible to environmental changes than adults. Adults however, have been shaped by the combined effects of genetic and environmental factors. Our previous study indicated that sAA response to gustatory stimulation might influence individual nutritional status in children; especially for those who traditionally fed on high-starch diet (Chen et al., 2015). Not surprisingly, in the present study we observed stronger sAA activity response to gustatory stimulation in children compared with adults.

In the present study, we observed an average number of eight copies of *AMY1* compared to previous studies of low-starch populations with average of 4.4 (Mandel et al., 2010) and 6.11 (Perry et al., 2007) copies. We also found that sAA levels have high variability among individuals. Mandel et al. found that the *AMY1* copy number was positively correlated with sAA concentration and activity, which in turn contributed to individual variations in the oral perception of starch viscosity and dietary starch intake (Mandel et al., 2010). More interestingly, Perry et al. found that individuals who historically consumed high starch diet, had on average more copies of *AMY1* gene than populations that traditionally fed on low-starch diet. These authors have argued that the *AMY1* gene copy number has been subject to positive selection in some high-starch populations but has evolved neutrally in low-starch populations (Perry et al., 2007). Thus, we considered that a significant proportion of our participants' ancestors may have undergone appositive selection for an increased copy number of the *AMY1* gene, rather than evolve neutrally.

Additionally, sAA activity was influenced by *AMY1* copy number and sAA protein expression. In order to assess the roles played by sAA protein expression, glycosylated modification, and *AMY1* genetic polymorphism in the age difference of sAA activity and sAA responses to citric acid stimulation between children and adults, we analyzed the correlations among sAA activity, sAA amount, and *AMY1* copy number in basal and stimulated saliva. Our findings showed that total sAA amount, glycosylated sAA amount, and *AMY1* copy number × total sAA amount were all positively correlated with sAA activity in children and adults. Interestingly, we observed correlation r between glycosylated sAA amount and sAA activity increased from 0.362 to 0.595 after citric acid stimulation in adults. This might probably due to the fact that the parotid gland secreted more glycosylated sAA after stimulation and that glycosylated sAA had higher enzymatic activity compared with non-glycosyalted sAA, which were also discussed above. Furthermore, more glycosylated sAA led to higher sAA activity. To our surprise, we observed that correlation r between glycosylated sAA amount and sAA activity decreased from 0.598 to 0.448 in children after stimulation, and that correlation r between total sAA amount and sAA activity also decreased from 0.687 to 0.478. This can't be explained by our observations that both total and glycosylated sAA amounts significantly increased after stimulation in children. However, we argue that this might be related to an increasing amount of glycosylation-incomplete sAA in stimulated saliva of children. Liu et al. reported that the shown attenuated sAA activity responses were partially due to their incomplete glycosylation of sAA in stimulated saliva (Liu et al., 2007).

For children and adults, neither sAA activity nor total sAA amount was found to correlate with the *AMY1* copy number in basal or stimulated saliva however, positive correlation was observed between sAA activity and *AMY1* copy number in unstimulated saliva of adults. This may suggest that *AMY1* copy number has little role in influencing acute sAA activity responses to citric acid stimulation; and again indicates high variability of sAA levels among or even within individuals. However, we expected a positive interaction between *AMY1* copy number and total sAA amount in influencing sAA activity. In line with our expectations, we found that *AMY1* copy number × total sAA amount showed positive correlations with sAA activity before and after stimulation in both groups, and that the correlation r between *AMY1* copy number × total sAA amount and sAA activity was higher than that between *AMY1* copy number, total sAA amount, and sAA activity. This clearly demonstrates a positive interaction between *AMY1* copy number and total sAA amount.

One potential limitation of this study was that we used citric acid to stimulate saliva secretion. Citric acid may interfere with saliva during sampling and thus affect assay results of sAA activity because it changes pH value of the stimulated saliva. Regarding this point, we had trained participants before sample collection as described in the methods, and sample collection was done under supervision. As a quality control, we measured pH of saliva samples, and saliva samples would be discarded if its pH value was less than 6.4, which was considered the lower limit of saliva pH value. We reported that no salvia samples in the present study were less than 6.4 as for pH value. Hence, citric acid was also a reliable stimulus if participants had been well trained and supervised in the collection procedure. However, future research could examine whether other gustatory or mechanical stimuli also have similar results across different age groups.

In summary, we reported for the first time that total sAA amount, glycosylated sAA amount, and an interaction between *AMY1* copy number and total sAA amount played crucial roles in influencing the age related differences of sAA activity before and after citric acid stimulation in children and adults. Besides, sAA secretion patterns might be different between children and adults, which might due to the developmental differences of salivary gland functions and energy demand, especially for those who traditionally fed on starch-based foods. We argue that sAA glycosylated levels might play a more important role in determining sAA activity than total sAA amounts do. Finally, the *AMY1* copy number might influence, but did not determine sAA levels before and after citric acid stimulation in children and adults.

## Author contributions

ZMY and LHC wrote the manuscript; ZMY and WWC were the principal investigators and together contributed to the development of the overall research plan, study protocol, and oversight, and analyzed the data; MZ, JL, and LHC were responsible for participant recruitment and saliva collection. JZ, JL, and LHC performed the Western Blotting and qPCR experiments; XRY performed the enzymatic activity assay. All authors revised the manuscript and approved the final version.

### Conflict of interest statement

The authors declare that the research was conducted in the absence of any commercial or financial relationships that could be construed as a potential conflict of interest.
